# The extent and magnitude of islet T cell infiltration as powerful tools to define the progression to type 1 diabetes

**DOI:** 10.1007/s00125-023-05888-6

**Published:** 2023-03-08

**Authors:** Paola S. Apaolaza, Diana Balcacean, Jose Zapardiel-Gonzalo, Teresa Rodriguez-Calvo

**Affiliations:** 1 Institute of Diabetes Research, Helmholtz Zentrum München, German Research Center for Environmental Health, Munich-Neuherberg, Germany; 2 German Center for Diabetes Research (DZD), Neuherberg, Germany; 3 Present Address: Novartis Pharma Stein, Stein, Switzerland

**Keywords:** Cell density, Image analysis, Infiltration, Insulitis, Islets, Pancreas, Pathology, T cells, Type 1 diabetes

## Abstract

**Aims/hypothesis:**

Insulitis is not present in all islets, and it is elusive in humans. Although earlier studies focused on islets that fulfilled certain criteria (e.g. ≥15 CD45^+^ cells or ≥6 CD3^+^ cells), there is a fundamental lack of understanding of the infiltration dynamics in terms of its magnitude (i.e. how much) and extent (i.e. where). Here, we aimed to perform an in-depth characterisation of T cell infiltration by investigating islets with moderate (1–5 CD3^+^ cells) and high (≥6 CD3^+^ cells) infiltration in individuals with and without type 1 diabetes.

**Methods:**

Pancreatic tissue sections from 15 non-diabetic, eight double autoantibody-positive and ten type 1 diabetic (0–2 years of disease duration) organ donors were obtained from the Network for Pancreatic Organ Donors with Diabetes, and stained for insulin, glucagon, CD3 and CD8 by immunofluorescence. T cell infiltration was quantified in a total of 8661 islets using the software QuPath. The percentage of infiltrated islets and islet T cell density were calculated. To help standardise the analysis of T cell infiltration, we used cell density data to develop a new T cell density threshold capable of differentiating non-diabetic and type 1 diabetic donors.

**Results:**

Our analysis revealed that 17.1% of islets in non-diabetic donors, 33% of islets in autoantibody-positive and 32.5% of islets in type 1 diabetic donors were infiltrated by 1 to 5 CD3^+^ cells. Islets infiltrated by ≥6 CD3^+^ cells were rare in non-diabetic donors (0.4%) but could be found in autoantibody-positive (4.5%) and type 1 diabetic donors (8.2%). CD8^+^ and CD8^−^ populations followed similar patterns. Likewise, T cell density was significantly higher in the islets of autoantibody-positive donors (55.4 CD3^+^ cells/mm^2^) and type 1 diabetic donors (74.8 CD3^+^ cells/mm^2^) compared with non-diabetic individuals (17.3 CD3^+^ cells/mm^2^), which was accompanied by higher exocrine T cell density in type 1 diabetic individuals. Furthermore, we showed that the analysis of a minimum of 30 islets and the use of a reference mean value for T cell density of 30 CD3^+^ cells/mm^2^ (the 30–30 rule) can differentiate between non-diabetic and type 1 diabetic donors with high specificity and sensitivity. In addition, it can classify autoantibody-positive individuals as non-diabetic or type 1 diabetic-like.

**Conclusions/interpretation:**

Our data indicates that the proportion of infiltrated islets and T cell density change dramatically during the course of type 1 diabetes, and these changes can be already observed in double autoantibody-positive individuals. This suggests that, as disease progresses, T cell infiltration extends throughout the pancreas, reaching the islets and exocrine compartment. While it predominantly targets insulin-containing islets, large accumulations of cells are rare. Our study fulfils the need to further understand T cell infiltration, not only after diagnosis but also in individuals with diabetes-related autoantibodies. Furthermore, the development and application of new analytical tools based on T cell infiltration, like the 30–30 rule, will allow us to correlate islet infiltration with demographic and clinical variables with the aim of identifying individuals at the very early stages of the disease.

**Graphical abstract:**

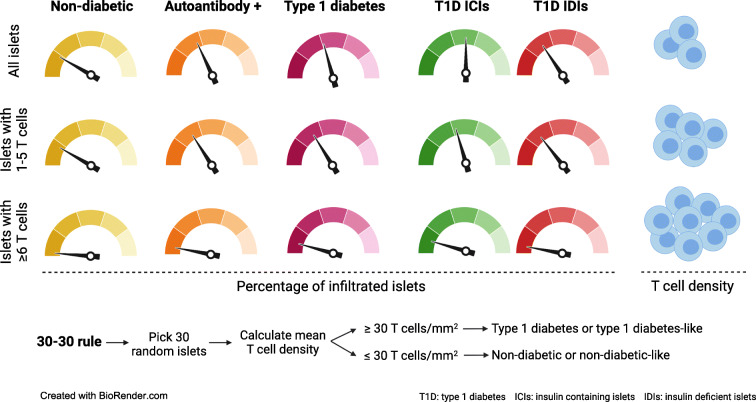

**Supplementary Information:**

The online version of this article (10.1007/s00125-023-05888-6) contains peer-reviewed but unedited supplementary material.

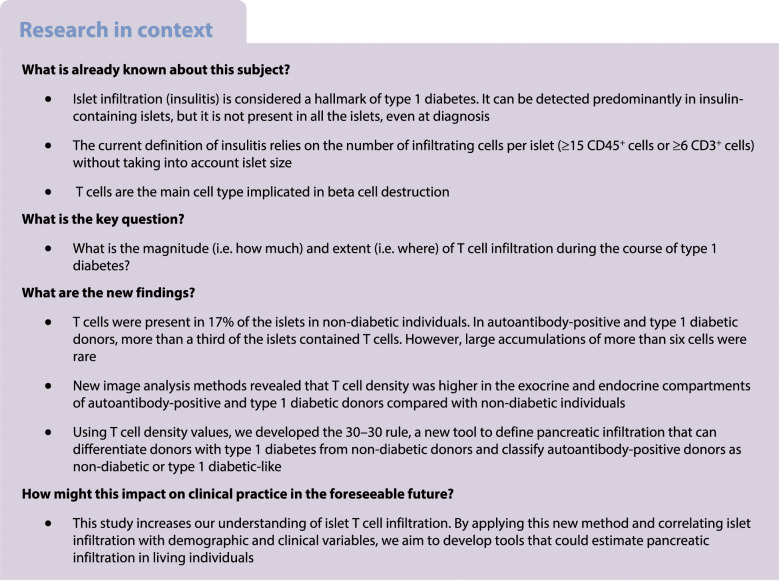



## Introduction

Insulitis was first observed more than 100 years ago and it is now considered a hallmark of type 1 diabetes [1]. It is described as a characteristic inflammatory infiltrate in the islets of Langerhans. For decades, the study of pancreas pathology, particularly insulitis, has been challenging due to limited access to human pancreas samples. Recently, several biobanks, such as the Network for Pancreatic Organ Donors with Diabetes (nPOD) [2, 3] and the Exeter Archival Diabetes Biobank (EADB) [4, 5], have provided new opportunities for investigating pancreatic immune infiltration and broadening our understanding of the pathogenesis of type 1 diabetes. Careful examination of human samples has shown that the inflammatory infiltrate in islets mainly consists of T cells, but macrophages and B cells are also common [6]. Insulitis is more common in younger individuals and in those with a short disease duration. Furthermore, it usually affects, but is not restricted to, islets that still retain insulin (insulin-containing islets; ICIs) [7, 8]. The aggressive, rapid destruction of islets in young children (<7 years old) is associated with the presence of a hyperimmune insulitic profile dominated by CD8^+^ cells, which are accompanied by CD4^+^ and CD20^+^ cells [9, 10]. Insulitis is more difficult to observe in adults, who seem to retain a larger proportion of ICIs [11, 12]. The proportion of donors with insulitis, of insulitic islets, and of ICIs, is extremely variable in both young and adult patients, and it is highly dependent on the disease duration. A common observation of prior studies is that not all the islets show high cell infiltration and not all ICIs exhibit insulitis.

Several definitions of insulitis have been proposed based on multiple immune cell populations, which has contributed to data heterogeneity and an overall lack of standardisation [6, 13, 14]. In 2013, a consensus definition was reached [15]. The definition included infiltration of cells into the islet periphery (peri-insulitis) and the islet parenchyma (intra-insulitis). A minimum of three islets infiltrated by ≥15 CD45^+^ cells/islet were required to be present in the pancreas. In addition, pseudoatrophic islets (insulin-deficient islets; IDIs) had to be present. In 2016, a new reference value for insulitis was proposed, which included islets infiltrated by six or more CD3^+^ cells immediately adjacent to or within the islet, in a minimum of three islets of a standard diameter (150 μm) per pancreas section [11]. Although these are useful reference values, none of these definitions were based on quantitative data involving accurate measurement of the size of each islet, which is highly variable in each pancreatic section. For example, a subset of large islets with a larger surface area might be infiltrated by a greater number of immune cells than smaller islets. Therefore, the relative importance of immune cell infiltration could vary when considering the size of the islet.

To normalise islet infiltration datasets and to allow comparison of multiple islets with different shapes, diameters or cell compositions, we and others have used islet density (number of cells/islet area in mm^2^; cells/mm^2^) [16, 17]. However, there are no comprehensive overviews that integrate the extent and magnitude of infiltration, and a new definition of insulitis based on cell density has not been developed. Using new image analysis tools, we performed a quantitative assessment of pancreatic T cell infiltration in pancreas sections from donors who were either non-diabetic, autoantibody-positive (AAb^+^), or had type 1 diabetes with a short disease duration (0–2 years). Furthermore, considering that relatively few islets satisfy the current definition of insulitis, we provide a new reference value based on cell density that can characterise pancreatic infiltration accurately in healthy and disease states.

## Methods

### Donors and samples

Pancreas sections from organ donors were collected through nPOD (www.jdrfnpod.org/). Frozen tissue sections were obtained from different regions of the pancreas (head, body and/or tail) from donors without diabetes (non-diabetic; *n=*15), donors who were positive for two autoantibodies (AAb^+^; *n=*8) and donors with recent-onset type 1 diabetes (*n=*10; disease duration of 0–2 years). The accession numbers available in the Resource Identification Portal (https://scicrunch.org/resources) and detailed information about each donor are provided in electronic supplementary material (ESM) Table 1. The inclusion criterion for this study was the presence of residual ICIs. Table 1 summarises the demographic information for each donor group. All experimental procedures were approved by the ethics committee at the Technical University of Munich (protocol #215/17 S) and Helmholtz Munich, Institute for Diabetes Research.

**Table 1:** Summarised donor demographic characteristics

Characteristic	Non-diabetic (*n*=15)	AAb^+^ (*n*=8)	T1D (*n*=10)	*p* value
Age, mean ± SD (years)	21.8 ± 9.5	25.6 ± 8.2	22.4 ± 6.5	0.58
Sex, *n* (%)				0.62
Female	8 (53.3)	3 (37.5)	6 (60)	
Male	7 (46.7)	5 (62.5)	4 (40)	
Ethnicity, *n* (%)				0.98
African American	3 (20)	2 (25)	3 (30)	
White	10 (66.7)	5 (62.5)	6 (60)	
Hispanic	2 (13.3)	1 (12.5)	1 (10)	
BMI, mean ± SD (kg/m^2^)	23.3 ± 4.9	28.2 ± 9.9	25.9 ± 7.7	0.29
Disease duration, mean ± SD (years)	–	–	1.05 ± 0.92	NA
C-peptide, mean ± SD (ng/ml)	7.8 ± 6.7	9.4 ± 7.7	1.4 ± 3.2	0.017
HbA_1c_ (mmol/mol)	36.5 ± 5.2	36.1 ± 3.3	86.4 ± 29.9	<0.001
HbA_1c_ (%)	5.5 ± 0.5	5.5 ± 0.3	10 ± 2.7	<0.001

Differences among the three groups were determined using an ordinary one-way ANOVA. Significant differences were found between donors with type 1 diabetes and the rest of the groups for C-peptide and HbA_1c_

NA, not analysed; T1D, type 1 diabetes

### Immunofluorescence

Frozen pancreatic sections were stained for insulin, glucagon, CD3 and CD8 by immunofluorescence. Tissue sections were fixed with 1% paraformaldehyde and blocked with 2% goat serum. Sections were incubated with primary antibodies for 1 h at room temperature. For detection, the sections were incubated for 1 h at room temperature with secondary antibodies. Sources and dilutions of primary and secondary antibodies are provided in ESM Table 2. Sections were counterstained with Hoechst 33342 (1:5000; Invitrogen, USA) for 8 min and mounted with Prolong Gold Antifade reagent (Invitrogen). The tissue sections were then scanned using an Axio Scan.Z1 slide scanner (Zeiss, Germany) and a ×20/0.8NA Plan-Apochromat (a=0.55 mm) objective.

### Image analysis

Blinded, manual analysis was performed to count the number of insulin- and glucagon-positive islets using ZEN Blue 2.3 software (Zeiss). QuPath v0.1.2 (University of Edinburgh, Division of Pathology, UK) [18], was used to determine the numbers of CD3^+^ and CD8^+^ cells and the percentage of infiltrated islets. An islet was considered infiltrated when at least one CD3^+^ cell was detected inside or immediately adjacent to the islet parenchyma. The image analysis pipeline has been described in Apaolaza et al [19]. Only islets formed by ≥30 cells were included to avoid possible detection errors resulting from small artefacts. Finally, all images were manually checked and corrected for any possible detection errors regarding islet boundaries, artefacts or the presence of autofluorescence.

Images for the CD3^+^ density threshold validation dataset were obtained from nPOD and consisted of whole-slide scanned images of frozen and paraffin-embedded pancreas samples stained for glucagon and CD3. In total, 25 donors (9 without diabetes and 16 with type 1 diabetes) were analysed. Donor IDs and basic demographic information are included in ESM Table 3.

### Statistical analysis

Continuous variables were compared using the Wilcoxon-signed rank test for paired samples. Differences among three or more groups were determined using the one-way ANOVA or Kruskal–Wallis test and *p* values were corrected for multiple comparisons using Dunn’s procedure. Spearman’s correlation analysis was used to evaluate the correlations among different variables. The significance level of the two-sided *p* values was 0.05 for all statistical tests. Statistical analyses were performed using R version 4.1.3 (www.r-project.org/) [20] and GraphPad Prism version 9.4.1 for Mac OS (GraphPad Software, www.graphpad.com). To assess the differences in islet area between ICIs and IDIs we used a linear mixed-effects model where the donor ID was included as a random effect. The analysis was performed using the package lme4 [21]. Receiver operating characteristic curves were plotted using the package pROC version 1.18.0 [22] and the bootstrapping procedures to determine the T cell density threshold were performed using the packages boot version 1.3-28 [23] and MonteCarlo version 1.0.6 (reference not available).

## Results

### An increasing proportion of islets are infiltrated during the course of type 1 diabetes

**Fig. 1:**
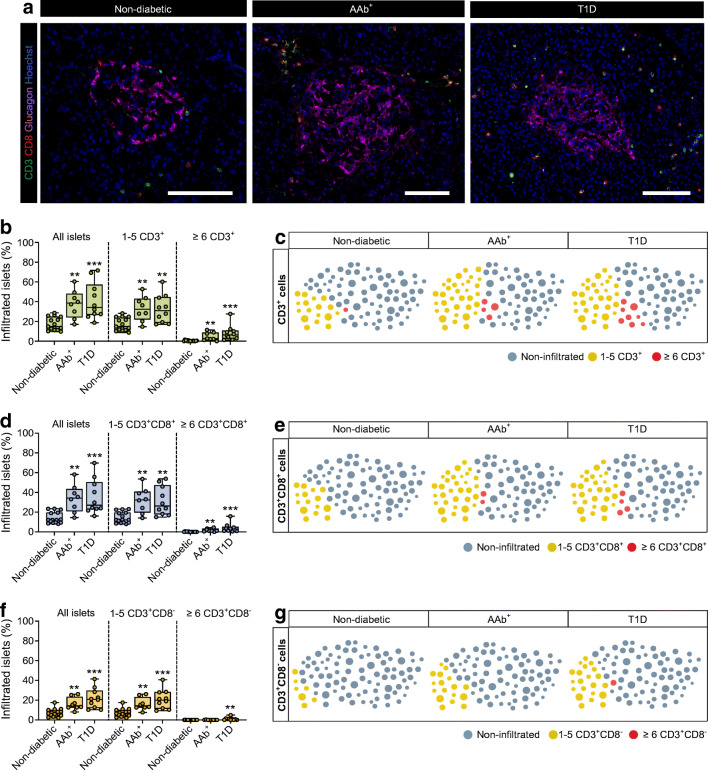
An increasing proportion of islets are infiltrated by T cells during the course of type 1 diabetes. (**a**) Representative immunofluorescence images of islets stained for glucagon (magenta) and of the surrounding exocrine tissue showing immune infiltration by CD3^+^ (green) and CD8^+^ (red) cells. Cell nuclei are shown in blue. Scale bar: 50 μm for non-diabetic and 100 μm for AAb^+^ and type 1 diabetic groups. (**b**, **d**, **f**) Box plots showing the median and the first and third quartiles for the percentages of islets infiltrated by CD3^+^ cells (**b**), CD3^+^CD8^+^ cells (**d**), and CD3^+^CD8^−^ cells (**f**). Each dot represents a donor. (**c**, **e**, **g**) Illustrations showing the proportions of islets infiltrated by CD3^+^ cells (**c**), CD3^+^CD8^+^ cells (**e**), and CD3^+^CD8^−^ cells (**g**), where each circle represents an islet (*n*=100, and the maximum is 100%). Non-infiltrated islets (blue), islets infiltrated by 1–5 CD3^+^ cells (yellow) and islets infiltrated by ≥6 CD3^+^ cells (red) are indicated. (**b**, **d**, **f**) Kruskal–Wallis test was performed. *p* values corrected for multiple comparisons using Dunn’s procedure. **p<0.01 and ***p<0.001. T1D, type 1 diabetes

Numerous studies have examined pancreatic T cell infiltration in diabetic and non-diabetic individuals. However, there is a lack of understanding of the extent of infiltration during disease progression, namely, how many islets are affected at a given time. T cell infiltration was quantified in 15 non-diabetic (*n*=5116 islets), eight double AAb^+^ (*n*=1537 islets) and ten type 1 diabetic (*n*=2008 islets) donors with short disease duration (0–2 years). Islets were identified by glucagon staining and T cells were quantified by CD3 and CD8 staining (Fig. 1a). Then, three groups of islets were established for subsequent analysis: (1) all islets; (2) islets infiltrated by 1–5 CD3^+^ cells; and (3) islets infiltrated by ≥6 CD3^+^ cells (ESM Table 4). First, the proportion of infiltrated islets was calculated (all islets group), which included islets infiltrated by any number of cells. In non-diabetic donors, a mean of 17.5% of all islets were infiltrated, compared with 37.5% and 40.7% in AAb^+^ and type 1 diabetic donors, respectively (Fig. 1b,c). Next, we quantified the proportion of islets infiltrated by 1–5 CD3^+^ cells, and the mean values were 17.1% in non-diabetic donors, 33% in AAb^+^ donors and 32.5% in type 1 diabetic donors (Fig. 1b,c). Furthermore, we determined the proportion of islets that would be considered to have insulitis, defined as islets infiltrated by ≥6 CD3^+^ cells. In total, 0.4% of islets were infiltrated in 8/15 non-diabetic donors, whereas 4.5% of the islets were infiltrated in 7/8 AAb^+^ donors. In type 1 diabetic donors, 8.2% of the islets were infiltrated by ≥6 CD3^+^ cells in 10/10 donors (Fig. 1b,c).

CD3^+^ cells were then subdivided into CD8^+^ and CD8^−^ cells for further quantification (ESM Table 4). CD8^+^ cells infiltrated 14.4% of all islets in non-diabetic donors compared with 33.6% and 35.3% in AAb^+^ and type 1 diabetic donors, respectively. Similar mean numbers were obtained for the percentage of islets infiltrated by 1–5 CD3^+^CD8^+^ cells (Fig. 1d,e). Then, the threshold of ≥6 CD3^+^CD8^+^ cells was applied. In type 1 diabetic donors, 4% of the islets showed infiltration, compared with 0.1% in non-diabetic donors, whereas the value in AAb^+^ donors was between the two groups (1.8%) (Fig. 1d,e). Furthermore, CD3^+^CD8^−^ T cells infiltrated 7.2% of all islets in non-diabetic donors, 16.2% in AAb^+^ donors and 21.7% in type 1 diabetic donors (Fig. 1f,g). The majority of these islets were infiltrated by 1–5 CD3^+^CD8^-^ cells. Conversely, few islets were infiltrated by ≥6 CD3^+^CD8^−^ cells (non-diabetic donors [0.007%] vs AAb^+^ [0.05%]). Although this proportion was low, it was significantly greater in type 1 diabetic donors (1%) (Fig. 1f,g).

### After disease onset, ICIs are preferentially infiltrated by a high number of T cells

**Fig. 2:**
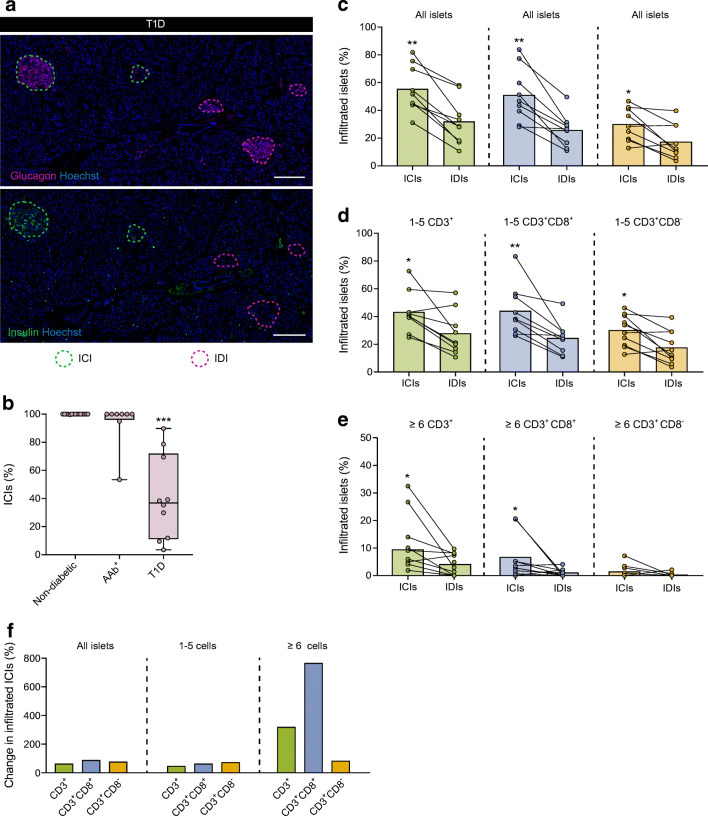
Insulin-containing islets from type 1 diabetic donors are preferentially infiltrated by a greater number of T cells. (**a**) Representative immunofluorescence images of consecutive sections from the same donor with type 1 diabetes showing IDIs identified by glucagon staining (magenta) and ICIs identified by insulin staining (green). The islet boundaries are highlighted by dashed lines in magenta and green, respectively. Cell nuclei are shown in blue. Scale bar: 200 μm. (**b**) Box plot showing the median and the first and third quartiles of the percentage of ICIs. Each dot represents a donor. (**c**–**e**) Bar graphs (mean) and individual paired values for the percentages of infiltrated ICIs and IDIs (all islets) (**c**), islets infiltrated by 1–5 CD3^+^ cells (**d**) and islets infiltrated by ≥6 CD3^+^ cells (**e**) in type 1 diabetic donors. (**f**) Percentage of change in the proportion of infiltrated islets in ICIs compared with that in IDIs for all islets, islets infiltrated by 1–5 cells and islets infiltrated by ≥6 cells. Data are shown for CD3^+^ cells (green), CD3^+^CD8^+^ cells (blue) and CD3^+^CD8^−^ cells (orange). (**c**, **f**) Data corresponds to the analysis of ICIs and IDIs from type 1 diabetic donors only. (**b**) Kruskal–Wallis test was performed. *p* values corrected for multiple comparisons using Dunn’s procedure. (**c**–**e**) Wilcoxon-signed rank test for paired samples was performed. *p<0.05, **p<0.01 and ***p<0.001. T1D, type 1 diabetes

In our cohort, the proportion of ICIs was significantly lower in type 1 diabetic donors (40.5%) than in AAb^+^ (93.5%) and non-diabetic (100%) donors (Fig. 2a,b). The percentage of remaining ICIs was not correlated with disease duration or age (data not shown). To understand whether the presence or absence of insulin influences T cell infiltration, we analysed a comparable number of ICIs (*n=*716) and IDIs (*n=*750) from type 1 diabetic donors (ESM Table 4). We found that ICIs were preferentially infiltrated independently of the immune marker used to define the T cell population: 55.4% of ICIs vs 34% of IDIs for CD3^+^ cells; 51.5% of ICIs vs 27.3% of IDIs for CD3^+^CD8^+^ cells; and 31% of ICIs vs 17.5% of IDIs for CD3^+^CD8^−^ cells (Fig. 2c). Similar results, albeit with lower percentages, were obtained for the proportion of islets infiltrated by 1–5 and ≥6 cells (Fig. 2d,e). In this analysis, individual islets could be infiltrated by both CD8^+^ and CD8^−^ cells. Side-by-side comparisons of the percentage of infiltrated islets in non-diabetic, AAb^+^, type 1 diabetic (ICIs + IDIs), type 1 diabetic ICIs, and type 1 diabetic IDIs are shown in ESM Fig. 1.

To examine the differences in the extent of infiltration between ICIs and IDIs further, the percentage of change in the proportion of infiltrated islets in ICIs relative to IDIs was calculated. The proportion of infiltrated islets was 62.7% higher for CD3^+^, 88.5% higher for CD3^+^CD8^+^ and 76.8% higher for CD3^+^CD8^−^ cells in ICIs relative to IDIs (Fig. 2f). When we applied the threshold of 1–5 cells, we obtained similar results to that for all islets (45% for CD3^+^, 61.1% for CD3^+^CD8^+^ and 71.5% higher for CD3^+^CD8^−^ cells in ICIs relative to IDIs). Furthermore, when we applied the threshold of ≥6 cells, the proportion of infiltrated islets was 316.3% higher for CD3^+^, 760.1% higher for CD3^+^CD8^+^ and 80.6% higher for CD3^+^CD8^−^ cells in ICIs relative to IDIs (Fig. 2f).

### T cell density increases during the course of type 1 diabetes

**Fig. 3:**
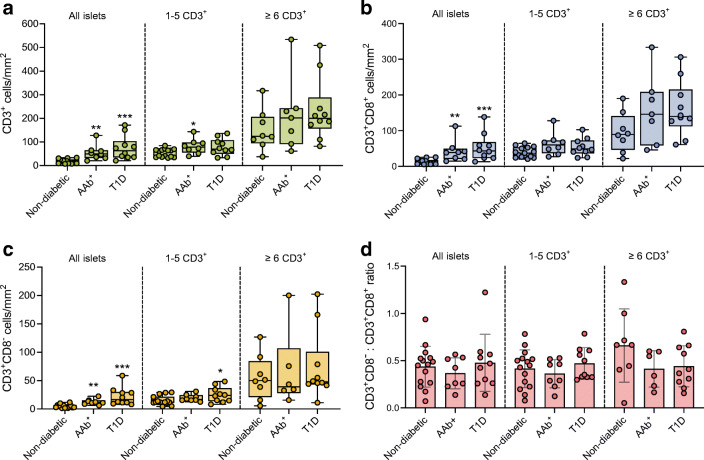
T cell density increases during the course of type 1 diabetes. (**a**–**c**) Box plots showing the median and first and third quartiles for the densities of CD3^+^ cells (**a**), CD3^+^CD8^+^ cells (**b**) and CD3^+^CD8^−^ cells (**c**) for all islets, islets infiltrated by 1–5 cells and islets infiltrated by ≥6 CD3^+^ cells. (**d**) Bar graph showing the mean CD3^+^CD8^−^:CD3^+^CD8^+^ ratio. Each dot represents a donor. (**a**–**d**) Kruskal–Wallis test was performed. *p* values corrected for multiple comparisons using Dunn’s procedure. *p<0.05, **p<0.01 and ***p<0.001. T1D, type 1 diabetes

A lingering issue in the analysis of pancreatic immune infiltration is how to account for islet size. In our cohort, there was a weak to moderate correlation between the number of infiltrating CD3^+^ cells and islet area (*r*=0.35, *p*<0.0001, data not shown). There were no statistically significant differences in the mean area of the islets between non-diabetic (0.019 mm^2^), AAb^+^ (0.021 mm^2^) and type 1 diabetic (0.021 mm^2^) donors (data not shown). To analyse the extent of infiltration and to consider the size of the islets, we calculated T cell density (number of T cells/mm^2^) for each islet (ESM Table 4). Among all islets analysed, the CD3^+^ density (CD3^+^ cells/mm^2^) was significantly greater in type 1 diabetic donors (74.8 CD3^+^ cells/mm^2^) than in AAb^+^ (55.4 CD3^+^ cells/mm^2^) and non-diabetic (17.3 CD3^+^ cells/mm^2^) donors (Fig. 3a). When we applied the thresholds of 1–5 CD3^+^ cells and ≥6 cells CD3^+^, we observed higher T cell densities in the AAb^+^ and type 1 diabetic groups compared with non-diabetic donors (Fig. 3a). However, this difference did not reach statistical significance. The CD3^+^ T cell densities for each islet, donor and reference value are shown in ESM Fig. 2.

Similarly, when these analyses were applied to CD3^+^CD8^+^ cells, we found significant differences between type 1 diabetic and non-diabetic donors for all islets (Fig. 3b, ESM Table 4). In islets infiltrated by 1–5 and by ≥6 CD3^+^ cells, CD3^+^CD8^+^ T cell density in AAb^+^ and type 1 diabetic donors was higher than in non-diabetic donors, although this difference did not reach statistical significance. CD3^+^CD8^−^ cell density showed similar results and was significantly greater in AAb^+^ and type 1 diabetic donors than in non-diabetic donors (Fig. 3c). When we used the reference of 1–5 CD3^+^ cells, only the difference between type 1 diabetic and non-diabetic donors remained statistically significant, whereas it was not significantly different for the proportion of islets infiltrated by ≥6 CD3^+^ cells (Fig. 3c). Last, to understand possible differences in the number of CD3^+^CD8^−^ and CD3^+^CD8^+^ cells, the ratio between these two cell types was calculated, but no significant differences were found (Fig. 3d).

### In donors with type 1 diabetes, T cell density is high in the remaining insulin-containing islets, but a small proportion of insulin-deficient islets show high infiltration

**Fig. 4:**
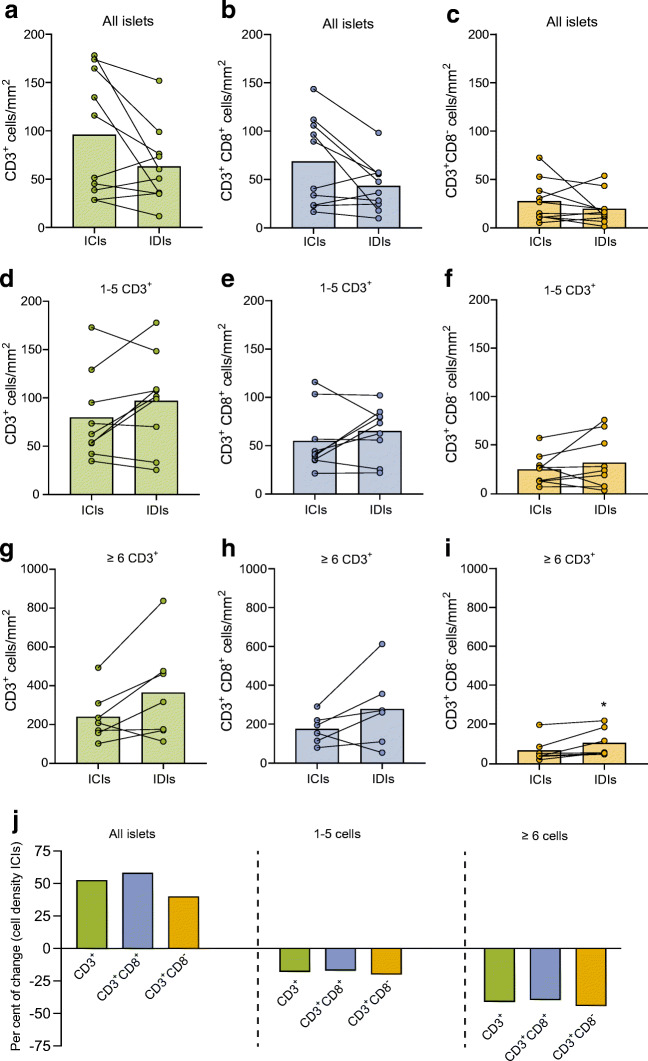
In type 1 diabetic donors, T cell density is higher in the remaining ICIs, but a few IDIs are highly infiltrated. (**a**–**i**) Bar graphs (mean) and individual paired values for the cell densities of ICIs and IDIs infiltrated by CD3^+^ cells (all islets) (**a**–**c**) by 1–5 CD3^+^ cells (**d**–**f**) and by ≥6 CD3^+^ cells (**g**–**i**) in type 1 diabetic donors. Data are shown for CD3^+^ cells (green) (**a**, **d**, **g**), CD3^+^CD8^+^ cells (blue) (**b**, **e**, **h**) and CD3^+^CD8^−^ cells (orange) (**c**, **f**, **i**). (**j**) Percentage of change in the T cell density in ICIs compared with that in IDIs for all islets, islets infiltrated by 1–5 cells and islets infiltrated by ≥6 cells. (**a**–**i**) Wilcoxon-signed rank test for paired samples was performed. *p<0.05

To determine whether the presence of insulin is a defining factor for increased cell density after diagnosis, T cell densities for ICIs and IDIs were compared within the same individuals in the type 1 diabetes group. First, the size of the islets was measured. IDIs were significantly smaller than ICIs (0.014 mm^2^ vs 0.027 mm^2^, *p<*0.0001). When all islets were included in the analysis, although not significant (*p*=0.06), T cell density in ICIs (*n=*712) was higher than in IDIs (*n=*750) (ESM Table 4) (Fig. 4a). Conversely, when we focused on islets with 1–5 CD3^+^ cells, T cell density was lower in ICIs (*n=*290) than in IDIs (*n=*176) (Fig. 4d). The same pattern was found when we focused on islets with ≥6 CD3^+^ cells for ICIs (*n=*87) and IDIs (*n=*28) (Fig. 4g). Similar results were obtained for CD3^+^CD8^+^ (Fig. 4b,e,h) and CD3^+^CD8^–^ (Fig. 4c,f,i) cell populations. Side-by-side comparisons of cell densities in non-diabetic, AAb^+^, type 1 diabetic (ICIs + IDIs), type 1 diabetic ICIs, and type 1 diabetic IDIs are shown in ESM Fig. 3.

Next, we calculated the percentage of change in the T cell density in ICIs relative to IDIs. In type 1 diabetic donors, the CD3^+^, CD3^+^CD8^+^ and CD3^+^CD8^–^ cell densities were increased by 52.5%, 58.18% and 40.12%, respectively, in ICIs relative to IDIs for all islets (Fig. 4j). Conversely, when we analysed islets with 1–5 CD3^+^ cells, the CD3^+^, CD3^+^CD8^+^ and CD3^+^CD8^–^ cell densities decreased by 7.4%, 16.4% and 19.4%, respectively, in ICIs relative to IDIs. Moreover, for ≥6 CD3^+^ cells, there was a decrease in cell density of 40.6% for CD3^+^, 39.2% for CD3^+^CD8^+^ and 43.8% for CD3^+^CD8^–^ (Fig. 4j).

### T cells infiltrate the exocrine pancreas in higher numbers in donors with type 1 diabetes than in autoantibody-positive or non-diabetic donors

**Fig. 5:**
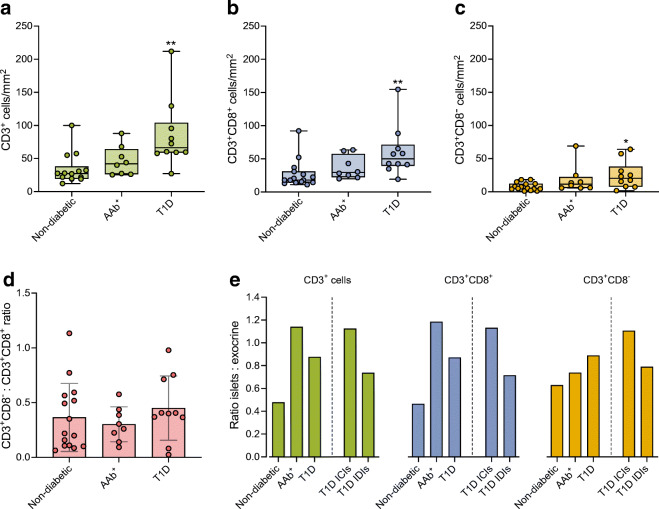
Greater numbers of T cells infiltrate the exocrine pancreas in type 1 diabetic donors compared with autoantibody-positive and non-diabetic donors. (**a**–**c**) Box plots showing the median and the first and third quartiles for the exocrine densities of CD3^+^ cells (**a**), CD3^+^CD8^+^ cells (**b**) and CD3^+^CD8^−^ cells (**c**). Each dot represents a donor. (**d**) Bar graph showing the mean CD3^+^CD8^−^:CD3^+^CD8^+^ ratio in the exocrine tissue. (**e**) Bar graph showing the mean ratio of CD3^+^ cell density between the islets and exocrine tissue for the non-diabetic, AAb^+^, and type 1 diabetic groups, and for ICIs and IDIs from type 1 diabetic donors. Data are shown for CD3^+^ cells (green), CD3^+^CD8^+^ cells (blue) and CD3^+^CD8^−^ cells (orange). (**a**–**d**) Kruskal–Wallis test was performed. *p* values corrected for multiple comparisons using Dunn’s procedure. *p<0.05, **p<0.01. T1D, type 1 diabetes

To evaluate T cell infiltration outside the islets, the number of T cells located in the exocrine tissue were quantified by dividing the number of infiltrating cells by the exocrine tissue area (total area minus the endocrine area) (ESM Table 4). CD3^+^ cell density was significantly greater in type 1 diabetic donors than in non-diabetic donors (34.8 cells/mm^2^ non-diabetic vs 55.4 cells/mm^2^ AAb^+^ vs 85.4 cells/mm^2^ type 1 diabetic) (Fig. 5a). Similar data were obtained for the CD3^+^CD8^+^ and CD3^+^CD8^−^ cell populations (Fig. 5b,c). The CD3^+^CD8^−^ : CD3^+^CD8^+^ cell ratio did not differ significantly among the groups (0.36 non-diabetic vs 0.30 AAb^+^ vs 0.45 cells type 1 diabetic) (Fig. 5d).

To analyse exocrine and endocrine T cell infiltration further, the ratio of endocrine to exocrine CD3^+^ cell densities was calculated. The ratio was 0.5 for CD3^+^, 0.5 for CD3^+^CD8^+^ and 0.6 for CD3^+^CD8^−^ cells in non-diabetic donors (Fig. 5e). In AAb^+^ donors, this ratio exceeded 1 for CD3^+^ and CD3^+^CD8^+^ while it was 0.7 for CD3^+^CD8^−^ cell populations. Furthermore, in donors with type 1 diabetes, the ratio ICIs to exocrine T cell density exceeded 1 in all cell populations, whereas it was 0.7 for IDIs (Fig. 5e).

### A cell density threshold to define T cell infiltration in type 1 diabetes: the 30–30 rule

**Fig. 6:**
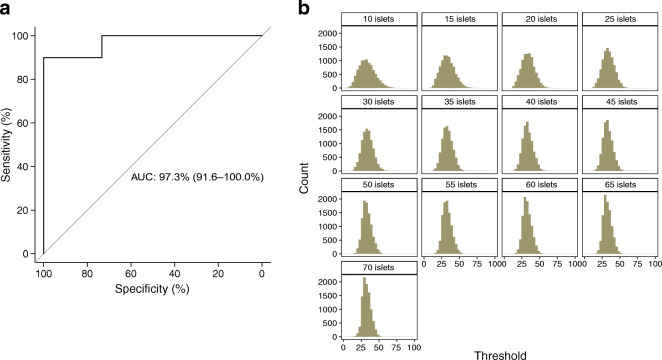
The 30–30 rule as a cell density threshold to define T cell infiltration in type 1 diabetes. (**a**) Receiver operating characteristic (ROC) curve for the classification of donors as non-diabetic or as type 1 diabetic based on CD3^+^ cell density. The AUC was calculated for the classifier using all available islets from each donor. The 95% CI is shown in brackets. (**b**) Distribution of the optimal threshold for the 10,000 iterations based on the number of randomly selected islets

To assess the validity of islet T cell density to differentiate between non-diabetic and type 1 diabetic donors, we calculated the mean CD3^+^ cell density value from each donor (defined as the sum of all islet-infiltrating CD3^+^ cells divided by the sum of all islet areas) and created a training dataset. Figure 6a shows the receiving operating characteristic curve for the binary classifier, for which the AUC was 97.3% (95% CI: 91.6%, 100%). Using the training data, an optimal threshold of 34.4 CD3^+^ cells/mm^2^ was obtained (see ESM results).

Up to this point, all the islets present in each section were included in the training dataset and used for the calculations. Then, the minimum number of analysed islets capable of differentiating non-diabetic and type 1 diabetic donors was calculated (see ESM results). The distribution of the optimal threshold values by the number of analysed islets is shown in Fig. 6b and the numerical results are shown in ESM Table 5. A threshold of 30 CD3^+^ cells/mm^2^ was chosen, which was then tested in an independent validation dataset, which included data from 25 donors (9 non-diabetic donors and 16 type 1 diabetic donors, described in methods). The computational analysis performed in the validation dataset confirmed the validity of the chosen T cell density threshold and showed that analysing 30 islets and applying a threshold of 30 CD3^+^ cells/mm^2^, the expected specificity and sensitivity were 87% (SD 5%) and 94% (SD 5%), respectively (Table 2).

**Table 2:** Validation of the CD3^+^ cell density threshold to differentiate non-diabetic and type 1 diabetic donors

*N*	Specificity (mean)	Specificity (SD)	Sensitivity (mean)	Sensitivity (SD)
10	0.83	0.09	0.85	0.07
15	0.85	0.07	0.89	0.06
20	0.85	0.06	0.91	0.06
25	0.86	0.06	0.93	0.05
30	0.87	0.05	0.94	0.05
35	0.87	0.04	0.95	0.04
40	0.87	0.04	0.96	0.04
45	0.88	0.04	0.96	0.04
50	0.88	0.03	0.97	0.03

The mean and SD for the sensitivity and specificity are shown. The number of islets was increased step-wise from 10 to 50. For each step, 10,000 iterations were performed, where islets were randomly selected, and the sensitivity and specificity were calculated

*N*, number of islets

### The 30–30 rule as a tool for classifying autoantibody-positive donors as non-diabetic or type 1 diabetic-like

To assess whether the threshold could be applied to AAb^+^ donors and whether it could classify them as non-diabetic or type 1 diabetic-like, CD3^+^ cell density data from the 8 AAb^+^ donors were analysed using the 30–30 rule (ESM Table 6 and ESM results). One donor was classified as non-diabetic in more than 90% of the iterations, whereas five were classified as type 1 diabetic-like in more than 95% of the iterations. Only two donors were classified as type 1 diabetic-like in about half of the iterations. To evaluate how the 30–30 rule performs in single AAb^+^ donors, available data from two donors were included. As opposed to the double AAb^+^ donors, the two single AAb^+^ donors were classified as type 1 diabetic-like in 14.5% and 17.8% of the iterations. The percentage of iterations in which a donor was classified as type 1 diabetic-like was not correlated with age, BMI, HbA_1c_, pancreas weight or time spent in the intensive care unit. Only the correlation with C-peptide approached significance (*r*=0.6, *p*=0.06).

## Discussion

Insulitis is a hallmark of type 1 diabetes. It is the visible evidence of immune cell infiltration in the islets, and often interpreted as proof of an immune attack on beta cells. Multiple studies have shown that the presence of insulitis at any given time is low, and it is rarely observed in more than a third of the islets in an individual [24]. However, there is a fundamental lack of understanding of the infiltration dynamics in terms of its magnitude (i.e. how much) and extent (i.e. where). Thus, we performed an in-depth characterisation of T cell infiltration by not only investigating islets with insulitis (≥6 T cells), but also islets infiltrated by fewer cells (1–5 T cells) to capture more subtle differences in islet infiltration. On the one hand, visualising the presence of T cells in a non-diabetic state could help us to understand the changes that occur during the early phases of type 1 diabetes. On the other hand, islets with low or high infiltration may represent different stages of the immune attack on beta cells during the course of type 1 diabetes.

In our cohort, 17% of the islets in non-diabetic individuals contained 1–5 CD3^+^ T cells, and this percentage almost doubled in AAb^+^ and type 1 diabetic donors. Therefore, we assume that, in a non-diabetic state, T cells might interact with islets without exerting deleterious effects on beta cells and contribute to the normal islet immune cell repertoire. When autoimmunity is triggered, a greater number of islets starts to be infiltrated, but its rate does not seem to change dramatically over time. Around 30% of the islets were infiltrated before and after onset, and fewer islets were infiltrated by a large number of cells. A limitation of the current study is that we focused on a predominantly adult population (mean age 23 ± 8.3 years old). As infiltration is often more prevalent and severe in younger individuals, similar studies may find a higher extent and magnitude of islet infiltration in younger donors.

Once T cells have found their target, we would expect that a massive number of cells would start to infiltrate the islets to eliminate the source of the specific antigen. However, the proportion of islets that have ≥6 T cells is low. The expression of programmed death-ligand 1 (PD-L1), an immune checkpoint inhibitor, might explain why certain islets can resist T cell-mediated apoptosis for a long time [25]. The proportion of highly infiltrated islets was more than ten times higher in AAb^+^ donors and 20 times higher in donors with type 1 diabetes, compared with non-diabetic donors. This indicates that although a small proportion of islets show high infiltration before and after onset, their steady increase during disease progression is a strong indicator of an ongoing pathogenic process. However, future studies should be directed towards defining the phenotype and function of these T cells, to shed light into their ‘physiological’ or ‘pathological’ role.

Previous studies by us and others have shown that CD8^+^ T cells are the main cell type that infiltrates islets [6, 17]. This was confirmed here, and we observed increasing percentages during disease progression. If we assume that the majority of CD3^+^CD8^−^ cells are CD4^+^ T cells, our data indicate that CD4^+^ cells follow a similar pattern to CD8^+^ cells. Islets infiltrated by CD4^+^ cells almost tripled in type 1 diabetic donors, suggesting that CD4^+^ T cells play a prominent role in this disease, even if their overall numbers are low. Another well-defined trait of insulitis is that it is predominantly found in islets that still contain insulin. The type 1 diabetic donors included in this study had a short disease duration (0–2 years), but there was marked heterogeneity in the proportion of remaining ICIs among donors. Notwithstanding this variation, infiltration mainly occurred in ICIs rather than in IDIs. ICIs infiltrated by ≥6 CD3^+^CD8^+^ T cells, were five times more frequent than IDIs; although almost 30% of the IDIs contained 1–5 CD3^+^ T cells, only 4% of IDIs were infiltrated by ≥6 cells. A caveat of the current study is that islets are three-dimensional structures and because we used thin tissue sections, we only observed a two-dimensional layer. The absence of visible insulin-containing beta cells did not preclude the existence of these cells in other planes of the islet. Therefore, it is possible that some IDIs were actually ICIs, with some remaining insulin-positive cells that could not be observed.

Overall, our data indicate that T cells infiltrate an increasing number of islets as type 1 diabetes progresses. Further analysis of T cell density followed a similar pattern. However, this was only true when we considered the mean of all islets (infiltrated and non-infiltrated). When we focused on infiltrated islets and discarded the non-infiltrated, the differences in T cell density started to become smaller or even change direction. This could be due to several reasons: (1) once an islet was classified as infiltrated, the magnitude of infiltration (or islet density) was not dramatically different between type 1 diabetic, AAb^+^ and non-diabetic groups; (2) since the islets are three-dimensional structures and we are only visualising a thin section, we might be underestimating the total number of infiltrating T cells; and (3) by focusing on infiltrated islets, we only considered 17% of the islets in non-diabetic and around 30% of the islets in AAb^+^ and type 1 diabetic donors, reducing the statistical power for detecting possible differences among these groups. Limiting the analysis to islets that satisfy the definition of insulitis made the decrease in the number of islets even more evident. Despite higher exocrine T cell density, infiltration in the islets of AAb^+^ donors and ICIs from type 1 diabetic donors was proportionally greater than in the exocrine tissue, indicating that immune cell attraction to the islets peaks before diagnosis, continues after onset and decreases once insulin is no longer present.

The aim of the current work was not only to characterise T cell infiltration in type 1 diabetes, but also to provide tools that could make this task easier for other investigators. Overall, we collected data from over 8000 islets across 33 donors. We concluded that a minimum of 30 islets and a reference T cell density value of 30 CD3^+^ T cells/mm^2^ provided good sensitivity and specificity, creating the 30–30 rule. The applicability of this rule expands beyond non-diabetic and type 1 diabetic donors, as proven by the classification of AAb^+^ individuals. Five out of eight double AAb^+^ donors were classified as having an infiltration profile close to that of type 1 diabetic donors, indicating that they would have progressed to disease. Conversely, one double AAb^+^ individual was classified as non-diabetic. This donor could have been in earlier disease stages or perhaps would not have progressed to type 1 diabetes. We acknowledge that the 30–30 rule has some limitations, as there will be some donors in whom the infiltration profile will not allow accurate classification. It is important to consider that the 30–30 rule was assessed in a defined cohort with a predominance of adult donors, and was applied to randomly selected islets out of the total number of islets present on a given tissue section.

In summary, we have shown that, during the course of the disease, an increasing number of T cells infiltrate an increasing proportion of islets, predominantly ICIs. A small but significant number of islets have a high T cell density, which likely reflects active beta cell destruction. Although T cell infiltration into the endocrine and exocrine compartments of the pancreas follows a similar path, high infiltration was clearest in the islets from double AAb^+^ donors and in ICIs from type 1 diabetic donors. Finally, we present new analytical tools for analysing T cell infiltration. By applying the 30–30 rule, we aim to increase our understanding of the early stages of type 1 diabetes, to identify individuals with active immune infiltration and to search for new biomarkers, with the ultimate goal to provide therapies to patients who will show greatest benefit in each disease stage.

## Supplementary information

ESM (PDF 1.67 MB)

## Data Availability

All data generated and analysed during this study are included in this published article (and its supplementary material files).

## Abbreviations

AAb^+^: Autoantibody-positive
ICIs: Insulin-containing islets
IDIs: Insulin-deficient islets
nPOD: Network for Pancreatic Organ Donors with Diabetes
