# Supplementary material for: The extent and magnitude of islet T cell infiltration as powerful tools to define the progression to type 1 diabetes
Source: Diabetologia. 2023 Mar 8;66(6):1129–41. doi: 10.1007/s00125-023-05888-6 (PMC10163126; doi:10.1007/s00125-023-05888-6)
Supplement: Supplementary file 1 — (PDF 1.67 MB) [file 125_2023_5888_MOESM1_ESM.pdf]

ESM results:

A cell density threshold to define T cell infiltration in type 1 diabetes: the 30-30 rule

To select an optimal $CD3^+$ density threshold, we calculated Youden's J statistic, which is the value that maximizes the distance to the identity (diagonal) line. The optimal threshold of 34.4 $CD3^+$ cells/mm^2 resulted in a sensitivity of 90% and a specificity of 100% for differentiating between non-diabetic and T1D donors. To measure the accuracy of this threshold, a bootstrap method was used. The islets of each donor were randomly sampled with replacement and the optimal threshold, sensitivity, and specificity were recalculated 10,000 times. The bias for the density threshold was −1.88 with a standard error of 4.03. The biases for the sensitivity and specificity were -0.01 and −0.07, with standard errors of 0.06 and 0.07, respectively.

To calculate the minimum number of analyzed islets capable of differentiating non-diabetic and T1D donors, 10 randomly selected islets per donor were used, and the number was increased in 5-islet steps up to 70 islets per donor (figure 6b). For each number of islets (10, 15, 20, etc.), the threshold calculation, sensitivity, and specificity were determined 10,000 times (table 2). A threshold of 30 $CD3^+$ cells/mm^2 was chosen.

Then, the 30-30 rule was applied to AAb^+ donors. For each donor, 10,000 iterations were performed, where 30 islets were randomly selected and the donor was classified as non-diabetic or T1D-like based on the mean density (ESM Table 6).

ESM Table 1 shows extended demographic and histological information, and pancreatic regions analyzed.

ID	RRIDs	Donor Type	Gender	Age	BMI	Duration	C-Peptide	Time ICU (days)	HLA transplant	Regions analyzed	Autoantibodies	Summarized histopathology
**6158**	SAMN15879214	Autoab Pos	Male	40.3	29.7	-	0.5	0.7	A*03/24, B*49/62, DR*04/13, DQB*06/07	Tail	GADA+ mIAA+	Ins+/Gluc+, range of sizes including large with regular spherical morphologies and expected numbers per region. Focal mild chronic inflammation with interstitial fibrosis, and few CD3+ infiltrating cells. Mild to moderate acinar fatty replacement.
**6167**	SAMN15879223	Autoab Pos	Male	37	26.3	-	5.4	1	A*01/03, B*60/52, DR*04/02, DQB*08/06	Tail	IA-2A+ ZnT8A	Ins+/Gluc+ islets, normal. No infiltrates. Mild acinar fat.
**6197**	SAMN15879253	Autoab Pos	Male	22	28.2	-	17.5	0.6	A*02/24, B*42/45, DR*07/18, DQB*02/04	Tail	GADA+ IA-2A+	Ins+/Gluc+ islets, plentiful. Insulitis (rare) with few pseudoatrophic islets. Islet hyperemia. Mild, multifocal CD3+ infiltrates within lobules and perilobular (pancreatitis).
**6267**	SAMN15879321	Autoab Pos	Female	23	23.5	-	16.6	3.6	A*01/11, B*39/60, DR*04/04, DQ*08/08	Tail	GADA+ IA-2A+	Ins+/Gluc+islets in normal numbers and density. Insulitis found in all regions. Islets appear well demarcated with some having fibrosis. Mild CD3+ infiltrates acinar region with mild exocrine atrophy
**6424**	SAMN15879477	Autoab Pos	Male	17.6	51.4	-	7	5.2	A*30/68, B*08/35, DR*17/04, DQ*02/08	Head, Body, Tail	GADA+ mIAA+	Ins+/Gluc+ islets, normal range of sizes and morphologies. Diffuse, mild to moderate increase in exocrine CD3/45+ cell numbers and perivascular or periductal infiltrates.Fewer than 3 insulin-negative islets observed so presence of pseudoatrophic islets not supported to date.
**6429**	SAMN15879482	Autoab Pos	Male	22.1	19.6	-	2.2	2.4	A*01/02, B*44/81, DR*103/17, DQ*02/05	Head, Body, Tail	GADA+ mIAA+	Ins+/Gluc+ islets, numerous with wide range sizes and reduced ratios beta/alpha cells. Most islets have irregular contours. No Ins- islets observed. Slight CD3+ perivascular and acinar infiltrates. Widespread, mild dilation of intralobular ducts. Mild-moderate exocrine degranulation not associated with CD45+ infiltrates.
**6450**	SAMN15879503	Autoab Pos	Female	22	24.4	-	5.5	1	A*01/33, B*08/65, DR*17/-, DQ*02/-	Tail	GADA+ ZnT8A+	Ins+/Gluc+ islets, abundant. Lobules with Ins- islets (pseudoatrophic). Focal islet hyperplasia, tail. Exocrine atrophy, highly variable, lobular corresponding with Ins- islets. Islet nuclear pleomorphism- moderate. Insulitis, multiple sections. High HLA, multiple sections.
**6505**	SAMN15879558	Autoab Pos	Female	20.59	22.4	-	20.8	1.6	A*02/03, B*35/44, DR*04/07, DQB*02/08	Body	GADA+ mIAA+	Ins+/Gluc+ islets for majority with 2 blocks containing Ins- islets. Islets are of regular sizes, shapes and numbers with numerous single beta- and alpha-cells. Moderate numbers of exocrine CD3+ cell with diffuse distributions.

**6104**	SAMN15879161	No diabetes	Male	41	20.5	-	20.5	1.8	A*29/68, B*39/44, C*12/16, DRB1*07/13, DQA1*01/02, DQB1*02/05	Tail	Negative	Ins+/Gluc+ islets. Mild adipose infiltration exocrine regions.
**6178**	SAMN15879234	No diabetes	Female	24.5	27.5	-	4.5	4.6	A*02/24, B*27/44, DR*04/15, DQ*06/03	Tail	Negative	Ins+/Gluc+ normal islets. No infiltrates.
**6232**	SAMN15879288	No diabetes	Female	14	20.8	-	19.5	0.6	A*02/24, B*07/44, DR*15/15, DQB*06/06	Body	Negative	Ins+/Gluc+ islets, numerous. No significant findings.
**6238**	SAMN15879294	No diabetes	Male	20	21.7	-	1.2	0.7	A*02/32, B*45/61, DR*11/18, DQ*04/07	Head, Body, Tail	Negative	Ins+/Gluc+ islets, normal numbers. No major infiltrates. Focal hemorrhage and compression of acinar tissue.
**6254**	SAMN15879310	No diabetes	Male	38	30.5	-	6.4	3.2	A*02/29, B*07/62, DR*07/17, DQ*02/02	Head, Body, Tail	Negative	Ins+/Gluc+ islets, normal range of sizes, morphologies, and numbers with larger sized islets more frequent in tail region. Moderate to severe fatty replacement of 1-2 lobules in a given section and overall high numbers of intralobular adipose cells. Rare foci of infiltrating polymorphonuclear cells that are highly Ki67+ (PH).
**6278**	SAMN15879332	No diabetes	Female	12	21.3	-	4.5	0.7	A*23/68, B*45/71, DR*11/12, DQ*05/07	Head, Body, Tail	Negative	Ins+/Gluc+ islets, numerous, medium to large sized with occasional cluster or single cells. Dilated vessels within islets. Acinar degranulation moderate.
**6331**	SAMN15879385	No diabetes	Female	27.1	24	-	3	4.8	A*26/34 B*35/49 DR*18/15 DQ*04/06	Tail	Negative	Ins+/Gluc+ islets, normal numbers and morphologies.
**6339**	SAMN15879393	No diabetes	Male	23.3	25	-	10.6	3.6	A*01/02, DRB1*03/10, DQA1*01/05:01, DQB1*02/05:01	Tail	Negative	Ins+/Gluc+ islets, normal morphologies and sizes including rare islets with reduced ratio beta to alpha cells. Very mild sporadic CD3+ periductal infiltrates. Mild to moderate interlobular fat.
**6366**	SAMN15879419	No diabetes	Female	21	20.5	-	0.4	0.5	A*02/24 B*27/48 DR*04/14 DQ*07/08	Tail	Negative	Ins+/Gluc+ islets with normal numbers and morphologies including single cells in acinar regions and ducts. Minimal exocrine infiltrates. Mild intralobular fibrosis, mild exocrine atrophy.
**6375**	SAMN15879428	No diabetes	Male	28.7	31.8	-	17.3	2.7	A*02/29, B*62/45, DR*04/14, DQ*08/05	Head, Body, Tail	Negative	Ins+/Gluc+ islets, normal numbers and sizes. Low with focally increased intralobular fat. Low exocrine infiltrates.
**6384**	SAMN15879437	No diabetes	Male	17	18.2	-	0.7	2.2	A*24/69, B*07/55, DR*11/15, DQ*06/07	Head, Body, Tail	Negative	Ins+/Gluc+ islets, normal. No significant exocrine abnormalities.
**6401**	SAMN15879454	No diabetes	Female	25	31.3	-	12.8	1.8	A*31/33, B*14/51, DR*07/13, DQ*02/06	Head, Body, Tail	Negative	Ins+/Gluc+ islets, within normal range of numbers, sizes, and morphologies. Low intralobular and interlobular fat.
**6406**	SAMN15879459	No diabetes	Male	6.9	16.8	-	4.1	1.6	A*02/11, B*44/51, DR*17/07, DQ*02/02	Head, Body, Tail	Negative	Ins+/Gluc+ islets within normal range of sizes, shapes and density per region.
**6413**	SAMN15879466	No diabetes	Female	10.1	19	-	5.3	1.1	A*01/02, B*08/51, DR*01/17, DQ*02/05	Tail	Negative	Ins+/Gluc+ islets, numerous with normal range of sizes and density. No insulin-negative islets observed.

**6482**	SAMN15879535	No diabetes	Female	18.7	20	-	7.5	0.9	A*03/11, B*38/50, DR*17/13, DQ*02/06	Tail	Negative	Ins+/Gluc+ islet, normal range of sizes, spherical morphology including elongated side views (cigar-shape), and densities. No significant lesions observed.
**6362**	SAMN15879415	T1D	Male	24.9	28.5	0	0.4	7.8	A*03/11, B*18/35, DR*01/03, DQ*02/05	Head, Body, Tail	GADA+	Ins+/Gluc+ islets, moderate reduction numbers of Ins+ islets. Many islets with abnormal morphologies (large, fusing smaller islets, irregular outlines, fibrosis). Insulitis in most Ins+ islets and small number of Ins- islets. Islet nuclear pleomorphism with variable hydropic degeneration. Mild to moderate acinar CD3+ infiltrates and variable fatty infiltration. Moderate exocrine atrophy with variable intralobular fibrosis.
**6367**	SAMN15879420	T1D	Male	24	25.7	2	0.4	3.7	A*02/29, B*18/44, DR*04/07, DQB*02/08	Head, Tail	Negative	Ins+/Gluc+ islets, numerous islets, some large with patchy loss of insulin+ islets, primarily head region. Mild to moderate exocrine adipose infiltrates and moderate peri-pancreatic adipose. Low numbers of CD3+ infiltrates exocrine and periductal regions.
**6371**	SAMN15879424	T1D	Female	12.5	16.6	2	0.1	4	A*01/68, B*08/65, DR*13/17, DQ*02/06	Tail	GADA+, IA-2A+, mIAA+, ZnT8A+	Ins+ (reduced numbers)/Gluc+ islets with widely varied sizes and overall reduced islet numbers. Insulitis: diffuse and/or aggregates of CD3+ cells at both insulin+ and insulin- islets. Moderate endocrine cell nuclear pleomorphism. Mild to moderate diffuse increase in exocrine CD3+ numbers.
**6380**	SAMN15879433	T1D	Female	11.6	14.6	0	0.2	2.4	A*33/68 B*71/53 DR*17/13 DQ*02/06	Head, Body, Tail	Negative	Ins+ (reduced)/Gluc+ islets, mostly small and reduced numbers. Pseudoatrophic islets (insulin-negative) are highly variable with expected heterogeneity in lobular distribution. Possible insulitis seen in both insulin+ and insulin- islets with rarely more than 6 CD3+ cells diffusely associated with islet periphery. CD45+ cells diffusely increased exocrine regions with mild to moderate increase in polymorphonuclear cells, particularly eosinophils. Most infiltrates appear peri-vascular. No fibrosis or exocrine atrophy.
**6396**	SAMN15879449	T1D	Female	17.1	22.6	2	0.1	10.8	A*23/24, B*44/49, DR*07/17, DQ*02/-	Tail	Negative	Ins+/Gluc+ islets (reduced numbers) with majority of insulin-negative (pseudoatrophic) islets in reduced overall numbers and range of small to moderate large sizes. Insulitis present including in insulin-negative islets of diffuse infiltration by few CD3+ cells or rarer small aggregate of CD3+ cells. Moderate diffuse increase in exocrine CD3+/CD45+ cell numbers with infrequent, small intralobular foci.
**6405**	SAMN15879458	T1D	Female	29.1	42.5	0.6	1.8	6	A*30/31, B*18/61, DR*04/17, DQ*02/08	Head, Body, Tail	GADA+, IA-2A+, ZnT8A+	Ins+/Gluc+ (majority) with normal sizes and morphologies. Ins-/Gluc+ islets as scattered small islets or within certain lobules. Insulitis present all regions. Variable peri-islet fibrosis.

**6414**	SAMN15879467	T1D	Male	23.1	28.4	0.43	0.2	2.4	A*01/23, B*07/08, DR*17/09, DQ*02/-	Tail	GADA+, mIAA+, ZnT8A+	Ins+/Gluc+ islets (majority), small to large sized with primarily oval morphologies; single endocrine cells exocrine regions. Ins- islets and insulitis (both aggregate and diffuse types) present. Exocrine atrophy is focally moderate with mild increase in perilobular fibrosis. Mild diffuse exocrine CD3+ infiltrates.
**6449**	SAMN15879502	T1D	Male	24	23	2	0.03	1.9	A*01/24, B*39/57, DR*01/01, DQ*05/05	Tail	IA-2A+, mIAA+, ZnT8A+	Ins+/Gluc+ islets, rare, irregular profiles (diffuse rather than spherical morphologies), decreased density (islet atrophy). Single beta-cells and clusters widely distributed. Insulitis, peri-islet and intra-islet, INS+ and INS- islets. Exocrine atrophy, moderate.
**6456**	SAMN15879509	T1D	Female	30.49	30.1	0	10.3	0.7	A*02/23, B*64/82, DR*04/13, DQ*08/06	Tail	GADA+, ZnT8A+	Ins+ islets, numerous with range in sizes and compact morphologies. Low numbers of insulin-negative islets and also insulitis present (aggregates and peri-islet). No exocrine infiltrates.
**6469**	SAMN15879522	T1D	Female	27.06	26.9	1.5	0.7	5.6	A*24/30, B*71/49, DR*04/04, DQ*08/08	Tail	GADA+	Ins+/Gluc+ islets are in majority though with varying numbers between pancreas regions and lobules within sections. Single beta-cells observed in exocrine regions or in slets or as clusters. Normal numbers of islets with regular morphology (spherical profiles). Insulitis present- <8 islets overall with peri-islet CD3+ cells in low numbers. Islet fibrosis, mild. Mild global CD3+ exocrine infiltrates all regions. Mild focal interstitial fibrosis, acinar atrophy, and duct obstruction.

ESM Table 1: Extended donor information. Autoab Pos, autoantibody positive donor. T1D, type 1 diabetic donor. Age and duration of disease are expressed in years unless otherwise indicated; BMI, body mass index; C-peptide is expressed in ng/ml; time ICU, time spent in the Intensive Care Unit; ZnT8A, zinc transporter 8 autoantibodies; IA-2A, intracytoplasmic domain of the tyrosine phosphatase IA-2 autoantibodies; mIAA, micro assay for insulin autoantibodies; GADA, glutamic acid decarboxylase 65 autoantibodies. – indicates not determined or not available.

Primary Antibodies	Manufacturer and catalogue number	Antibody dilution
Insulin	Agilent Technologies, A056401	1:140
Glucagon	Abcam, ab92517	1:400
Glucagon	R&D Systems, MAB1249	1:100
CD3	Agilent Technologies, M725429-2	1:300
CD8	BD Biosciences, 550372	1:100

Secondary Antibodies	Manufacturer and catalogue number	Antibody dilution
Goat anti-mouse IgG2a Alexa Fluor 488	Invitrogen, A-21131	1:1000
Goat anti-mouse IgG1 Alexa Fluor 555	Invitrogen, A-21127	1:1000
F(ab')2 fragment of goat anti-rabbit Alexa Fluor 647	Jackson ImmunoResearch, 111-606-144	1:800
F(ab')2 fragment of goat anti-rabbit Alexa Fluor 555	Invitrogen, A-21430	1:1000
Goat anti-guinea pig IgG (H+L) Alexa Fluor 488	Invitrogen, A-11073	1:1000

ESM Table 2: Primary and secondary antibodies, manufacturer, catalogue number and antibody dilution.

ESM Table 3 shows extended demographic and histological information, pancreatic regions analyzed and type of tissue section for the donors included in the T cell density validation dataset.

ID	RRIDs	Donor Type	Gender	Age	BMI	Duration	C-Peptide	Time ICU (days)	HLA transplant	Autoantibodies	Summarized histopathology	Regions analyzed	Type of tissue section
**6179**	SAMN15879235	No Diabetes	Female	20	20.7	-	2.74		A*02/24, B*15/27, DR*03/04, DQB*02/08	Negative	Ins+/Gluc+ islets, normal range of sizes, morphologies and density. No other significant abnormalities observed.	Tail	Paraffin
**6227**	SAMN15879283	No Diabetes	Female	17	26.4	-	2.75	10.1	A*02/03, B*60/62, DR*04/13, DQB*06/08	Negative	Ins+/Gluc+ islets, no abnormalities observed.	Tail	Paraffin
**6230**	SAMN15879286	No Diabetes	Male	16	19.4	-	5.22	1.1	A*23/32, B*44/49, DR*04/11, DQB*07/08	Negative	Ins+/Gluc+ islets, no abnormalities observed.	Tail	Paraffin
**6232**	SAMN15879288	No Diabetes	Female	14	20.8	-	19.5	0.6	A*02/24, B*07/44, DR*15/15, DQB*06/06	Negative	Ins+/Gluc+ islets, numerous. No significant findings.	Tail	Paraffin
**6235**	SAMN15879291	No Diabetes	Male	30	25.4	-	8.1	1.3	A*24/68, B*35/51, DR*04/17, DQB*02/08	Negative	Ins+/Gluc+ islets, numerous. No other major findings.	Tail	Paraffin
**6250**	SAMN15879306	No Diabetes	Male	40	27.9	-	7.31	0.7	A*01/32, B*08/44, DR*15/17, DQB*null/null	Negative	Ins+/Gluc+ islets present in normal numbers. No major abnormalities observed.	Tail	Paraffin
**6271**	SAMN15879325	No Diabetes	Male	17	24.4	-	11.47	-	A*02/null, B*27/44, DR*07/15, DQB*02/05	Negative	Ins+/Gluc+ islets, numerous, medium sized.	Tail	Paraffin
**6336**	SAMN15879390	No Diabetes	Female	14.3	28.9	-	7.87	2.1	A*01/26, B*13/38, DR*07/13, DQB*02/06	Negative	Ins+/Gluc+ islets, normal size range and numbers. Slight acinar fat.	Tail	Paraffin
**6353**	SAMN15879406	No Diabetes	Male	13	28.3	-	1.76	2	A*03/11, B*44/null, DR*04/null, DQB*07/08	Negative	Ins+/Gluc+ islets, Mild extra-pancreatic fat. Low acinar CD3+ cell numbers.	Tail	Paraffin
**6052**	SAMN15879109	T1D	Male	12	20.3	1	0.18	0.9	A*02/68, B*27/50, DR*09/16, DQB*05/09	IA-2A+, mIAA+	Ins+ (reduced numbers)/Gluc+ islets. Insulitis, especially peri-islet. Heterogeneous islet distribution and size. Very high islet Ki67+.	Tail	Frozen

**6084**	SAMN15879141	T1D	Male	14.2	26.3	4	<0.05	0.9	A*01/32, B*08/60, DR*17/04, DQB*02/08	mIAA+	Ins+/Gluc+ islets in lobules with moderate to severe adipose infiltration. Very irregular islets. Lobular adipose infiltration. Moderate islet nuclear pleomorphism. Mild exocrine, peri-adipocyte, and periductal CD3+ infiltrates.	Body	Frozen
**6087**	SAMN15879144	T1D	Male	17.5	21.9	4	<0.05	0.57	A*null/null, B*null/null, DR*04/17, DQB*02/08	mIAA+, ZnT8A+	Ins-/Gluc+ islets- reduced frequency. Mild, focal CD3+ islet infiltrates.	Tail	Paraffin
**6113**	SAMN15879170	T1D	Female	13.1	24.7	1.58	<0.05	4.18	A*02/03, B*08/44, DR*01/17, DQB*02/05	mIAA+	Ins+ (reduced)/Gluc+ islets. Insulitis. Lobular islet heterogenity. Islet atrophy with mild acinar atrophy (heterogeneous). Glucagon cells as single cells and small clusters.	Tail	Frozen
**6195**	SAMN15879251	T1D	Male	19.3	23.7	5	<0.05	0.54	A*03/03, B*07/15(62), DR*04/15, DQB*03(8)/06	GADA+, IA-2A+, mIAA+, ZnT8A+	Ins+/Gluc+ islets, in greatly reduced numbers, and single beta-cells in reduced numbers. Majority of islets are Gluc+ only (pseudoatrophic) with single alpha-cells and both islets, single cells are numerous while islet morphology is highly irregular in outlines. Insulitis is present in primarily INS- islets and is widespread throughout pancreas.	Tail	Frozen
**6198**	SAMN15879254	T1D	Female	22	23.1	3	<0.05	20.4	A*02/30, B*18/53, DR*04/11, DQB*null/null	GADA+, IA-2A+, mIAA+, ZnT8A+	Ins+/Gluc+ islets, lobular heterogeneity with small to medium sized islets of mostly spherical morphologies. Diffuse mild insulitis (increase in CD3+cells/islet rather than focal aggregates). Mild diffuse chronic inflammation with mild, multifocal exocrine CD3+ infiltrates and mild to moderate intralobular and perilobular fibrosis.	Tail	Frozen
**6211**	SAMN15879267	T1D	Female	24	24.4	4	<0.05	4.9	A*02/03, B*08/45, DR*04/12, DQB*07/08	GADA+, IA-2A+, mIAA+, ZnT8A+	Ins+/Gluc+ islets in reduced numbers with highly variable distribution between regions. Majority of islets are Ins-/Gluc+ with medium sizes, mostly spherical morphology and expected numbers per region.	Tail	Paraffin
**6212**	SAMN15879268	T1D	Male	20	29.1	5	<0.05	3	A*01/02, B*44/62, DR*07/07, DQB*02/02	mIAA+	Ins+(reduced)/Gluc+ islets. Insulitis- 1-2 islets, mild (>6 CD3+cells).	Tail	Frozen
**6224**	SAMN15879280	T1D	Female	21	22.8	1.5	<0.05	1.8	A*02/11, B*39/51, DR*01/04, DQB*null/null	Negative	Ins+ (reduced numbers and intensity)/Gluc+ islets with occasional insulitis (body, tail).	Tail	Frozen

**6228**	SAMN15879284	T1D	Male	13	17.36	0	0.1	9.5	A*23/68, B*60/44, DR*17/04, DQB*02/08	GADA+, IA-2A+, ZnT8A+	Ins+/Gluc+ islets of various morphologies including normal-appearing spherical, mild fibrosis with degeneration, and several islets with CD3+ insulitis as aggregates as well as infiltrating eosinophils. Some pseudoatrophic (glucagon+ only) islets. Very mild, multifocal exocrine atrophy. Mild, multifocal CD3+ mononuclear acinar infiltrates with focal accumulations (chronic inflammation).	Tail	Frozen
**6243**	SAMN15879299	T1D	Male	13	21.3	5	0.42	3.5	A*01/02, B*08/08, DR*17/17, DQB*02/02	mIAA+	Ins+/Gluc+ islets and single beta-cells (reduced numbers but present throughout all blocks), glucagon+ only islets (pseudoatrophic). Insulitis present. Variable mild fatty exocrine infiltration. Diffuse moderate CD3+ exocrine infiltrates.	Tail	Frozen
**6247**	SAMN15879303	T1D	Male	24	24.3	0.6	0.47	0.6	A*24/68, B*44/60, DR*04/11, DQB*null/null	mIAA+	Ins+ (reduced numbers)/Gluc+ islets. Insulitis-sparse. Most sections have insulin+ islets, insulitis still rarely seen and mostly diffuse throughout dispersed islets. Also focal acinar infiltrates around dispersed islets.	Tail	Frozen
**6268**	SAMN15879322	T1D	Female	12	26.6	3	0.05	2.8	A*02/68, B*18/60, DR*13/17, DQB*02/06	mIAA+	Ins+ (very rare)/Gluc+ islets, possibly reduced islet numbers but increased glucagon+ single cells. Insulitis present at insulin+ and insulin- islets.	Tail	Paraffin
**6324**	SAMN15879378	T1D	Male	29	26.2	2	<0.02	2.8	A*11/24, B*18/52, DR*11/15, DQB*07/06	GADA+, mIAA+	Ins+/Gluc+ islets, rare, Insulitis present, both focal and diffuse in Ins+ and Ins- islets with the majority of insulitic islets being Ins-. Moderate CD3+ infiltrates all regions with focal chronic inflammation.	Tail	Paraffin
**6337**	SAMN15879391	T1D	Female	20.6	17.9	5	<0.02	3.5	A*01/33, B*08/65, DR*01/17, DQB*02/05	mIAA+	Ins+ (rare)/Gluc+ islets, reduced numbers and sizes. Moderate acinar atrophy. Minimal exocrine CD3+ infiltrates.	Tail	Frozen

**6342**	SAMN15879396	T1D	Female	14	24.3	2	0.26	1.5	A*02/68, B*60/null, DR*01/04, DQB*08/05	IA-2A+, mIAA+	Ins+ (reduced numbers)/Gluc+, expected numbers, some with moderately increased sizes. Majority of islets are insulin- (pseudoatrophic) yet most blocks contain several insulin+ islets, with and without insulitis. Islet nuclear pleomorphism particularly in larger islets. CD3+/CD45+ infiltrates present in exocrine regions, mild to focally moderate throughout pancreas.	Tail	Paraffin

ESM Table 3: Extended information for the donors included in the T cell density validation dataset. Autoab Pos, autoantibody positive donor. T1D, type 1 diabetic donor. Age and duration of disease are expressed in years unless otherwise indicated; BMI, body mass index; C-peptide is expressed in ng/ml; time ICU, time spent in the Intensive Care Unit; ZnT8A, zinc transporter 8 autoantibodies; IA-2A, intracytoplasmic domain of the tyrosine phosphatase IA-2 autoantibodies; mIAA, micro assay for insulin autoantibodies; GADA, glutamic acid decarboxylase 65 autoantibodies. – indicates not determined or not available.

	CD3⁺			CD3+ CD8⁺			CD3⁺ CD8⁻		
	Non-diabetic	AAb+	T1D	Non-diabetic	AAb+	T1D	Non-diabetic	AAb+	T1D
Infiltrated islets (%) (all)	17.5 ± 6.6	37.5 ±14.2	40.7 ±18.7	14.4 ± 5.6	33.6 ± 14.1	35.3 ± 17.5	7.2 ± 4.0	16.2 ± 6.4	21.7 ± 10.1
Infiltrated islets (%) (1-5 cells)	17.1 ± 6.5	33.0 ± 12.6	32.5 ±14.2	14.3 ± 5.6	31.9 ± 12.9	31.3 ± 14.6	7.2 ± 4.1	16.1 ± 6.4	20.7 ± 9.7
Infiltrated islets (%) (≥6 cells)	0.4 ± 0.5	4.5 ± 4.2	8.2 ± 7.7	0.1 ± 0.2	1.8 ± 1.5	4.0 ± 4.6	0.007 ± 0.02	0.05 ± 0.1	1.0 ± 1.5
Infiltrated ICIs (%) (all)	NA	NA	55.4 ± 16.8	NA	NA	51.5 ± 18.8	NA	NA	31.0 ± 11.9
Infiltrated ICIs (%) (1-5 cells)	NA	NA	43.2 ± 14.8	NA	NA	43.2 ± 17.9	NA	NA	29.2 ± 11.7
Infiltrated ICIs (%) (≥6 cells)	NA	NA	12.2 ± 10.6	NA	NA	6.5 ± 7.9	NA	NA	1.5 ± 2.4
Infiltrated IDIs (%) (all)	NA	NA	34.0 ± 17.0	NA	NA	27.3 ± 12.9	NA	NA	17.5 ± 11.7
Infiltrated IDIs (%) (1-5 cells)	NA	NA	27.8 ± 15.8	NA	NA	24.0 ± 11.5	NA	NA	16.9 ± 12.2
Infiltrated IDIs (%) (≥6 cells)	NA	NA	4.2 ± 4.1	NA	NA	1.1 ± 1.4	NA	NA	0.3 ± 0.7
Cell density (cells/mm²) (all)	17.3 ± 9.5	55.4 ± 33.0	74.8 ± 50.6	12.4 ± 7.4	42.8 ± 30.8	52.6 ± 38.0	4.9 ± 3.1	12.5 ± 4.9	22.2 ± 15.7
Cell density (cells/mm²) (1-5 cells)	54.0 ± 17.2	81.6 ± 32.1	78.8 ± 35.1	39.1 ± 14.4	62.4 ± 31.3	53.7 ± 24.4	14.9 ± 7.9	19.3 ± 6.1	25.1 ± 13.6
Cell density (cells/mm²) (≥6 cells)	150.1 ± 86.4	215.6 ± 156.2	234.4 ± 133.0	95.0 ± 55.5	158.2 ± 98.0	160.4 ± 76.9	55.0 ± 39.4	67.0 ± 68.2	74.0 ± 60.9
Cell density ICIs (cells/mm²) (all)	NA	NA	95.9 ± 63.6	NA	NA	68.3 ± 45.7	NA	NA	27.6 ± 21.6
Cell density ICIs (cells/mm²) (1-5 cells)	NA	NA	79.7 ± 45.5	NA	NA	54.5 ± 32.4	NA	NA	25.1 ± 15.8
Cell density ICIs (cells/mm²) (≥6 cells)	NA	NA	239.1 ± 129.6	NA	NA	169.6 ± 71.9	NA	NA	69.5 ± 61.7
Cell density IDIs (cells/mm²) (all)	NA	NA	62.9 ± 40.2	NA	NA	43.2 ± 25.6	NA	NA	19.7 ± 16.3
Cell density IDIs (cells/mm²) (1-5 cells)	NA	NA	96.8 ± 49.2	NA	NA	64.7 ± 26.8	NA	NA	32.1 ± 27.4
Cell density IDIs (cells/mm²) (≥6 cells)	NA	NA	364.1 ± 253.1	NA	NA	256.2±190.3	NA	NA	107.8 ± 72.0
Cell density Exocrine (cells/mm²)	34.8 ± 22.6	55.4 ± 26.7	85.4 ± 52.0	26.9 ± 21.0	36.8 ± 17.5	60.4 ± 38.1	7.8 ± 5.8	18.6 ± 21.3	24.9 ± 20.8

ESM Table 4: Mean ± Standard Deviation of the percentage of infiltrated islets (all), percentage of islets infiltrated by 1-5 cells (1-5 cells), percentage of islets infiltrated by ≥6 cells (≥6 cells), cell density in all the islets (all), cell density in islets infiltrated by 1-5 cells (1-5 cells), cell density in islets infiltrated by ≥6 cells (≥6 cells), and exocrine cell density for $CD3^+$, $CD3^+CD8^+$, and $CD3^+CD8^-$ cell populations are shown. Cell density is expressed in cells/mm². NA: Not analyzed.

	THRESHOLD		SPECIFICITY		SENSITIVITY	
N	MEAN	SD	MEAN	SD	MEAN	SD
10	32.92	11.42	0.88	0.11	0.79	0.13
15	33.40	10.03	0.90	0.10	0.82	0.11
20	33.66	9.06	0.91	0.09	0.83	0.11
25	33.70	8.36	0.92	0.08	0.84	0.10
30	33.59	7.90	0.92	0.08	0.85	0.10
35	33.23	7.38	0.92	0.08	0.85	0.10
40	33.26	7.18	0.92	0.08	0.86	0.09
45	33.02	6.77	0.92	0.08	0.86	0.09
50	32.89	6.55	0.92	0.08	0.87	0.09
55	32.84	6.34	0.92	0.08	0.87	0.09
60	32.54	6.18	0.92	0.08	0.87	0.09
65	32.53	5.96	0.92	0.08	0.87	0.09
70	32.56	5.79	0.92	0.08	0.88	0.08

ESM Table 5: Calculation of the minimum number of islets capable of differentiating non-diabetic and T1D donors. The mean $CD3^{+}$ cell density value from each donor was used to create a T cell density threshold. Initially, 10 randomly selected islets per donor were used, and the number was increased in 5-islet steps up to 70 islets per donor. The mean and standard deviation for the threshold value, sensitivity, and specificity are shown for the indicated number of islets, and were calculated in 10,000 iterations. Islets were randomly selected from the training dataset. N, number of islets; SD, standard deviation.

Donor ID	# of AAb	T1D-like (% of iterations)
6158	2	96.7
6167	2	98.5
6197	2	100
6267	2	99.1
6400	1	14.5
6421	1	17.8
6424	2	53.3
6429	2	9.9
6450	2	98.3
6505	2	60.3

ESM Table 6: The 30-30 rule as a tool to classify AAb+ donors as non-diabetic- or T1D-like. To assess whether the threshold could be applied to AAb+ donors and whether it could classify them as non-diabetic or T1D-like, $CD3^{+}$ cell density data from 8 AAb+ donors were analyzed using the 30-30 rule. For each donor, 10,000 iterations were performed, where 30 islets were randomly selected and the donor was classified as non-diabetic or T1D-like based on the mean density. Donor IDs and percentage of iterations resulting in a type 1 diabetes-like classification are shown. AAb, autoantibodies.

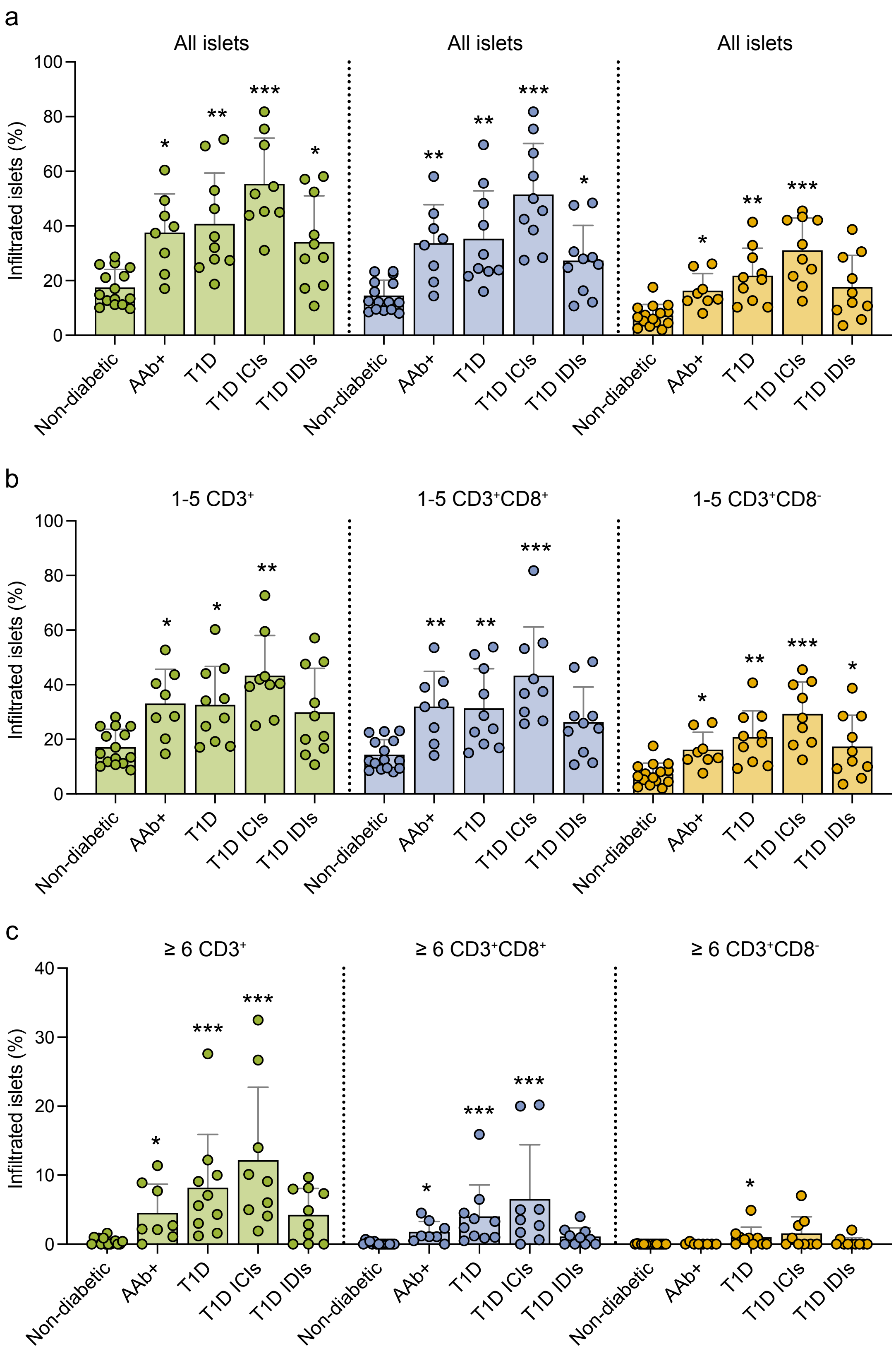

ESM fig. 1: Summarized data showing the proportion of infiltrated islets for each group. Bar graphs (mean + standard deviation) show the percentage of infiltrated islets for the non-diabetic, AAb+, and T1D groups, and for ICIs, and IDIs from T1D donors for the different cell thresholds: (a) all islets, (b) islets infiltrated by 1-5 cells, and (c) islets infiltrated by ≥6 cells. Data are shown for $CD3^+$ cells (green), $CD3^+CD8^+$ cells (blue), and $CD3^+CD8^-$ cells (orange). Kruskal–Wallis test in a-c was performed. *p*-values corrected for multiple comparisons using Dunn's procedure. $^*p < 0.05$, $^{**}p < 0.01$ and $^{***}p < 0.001$.

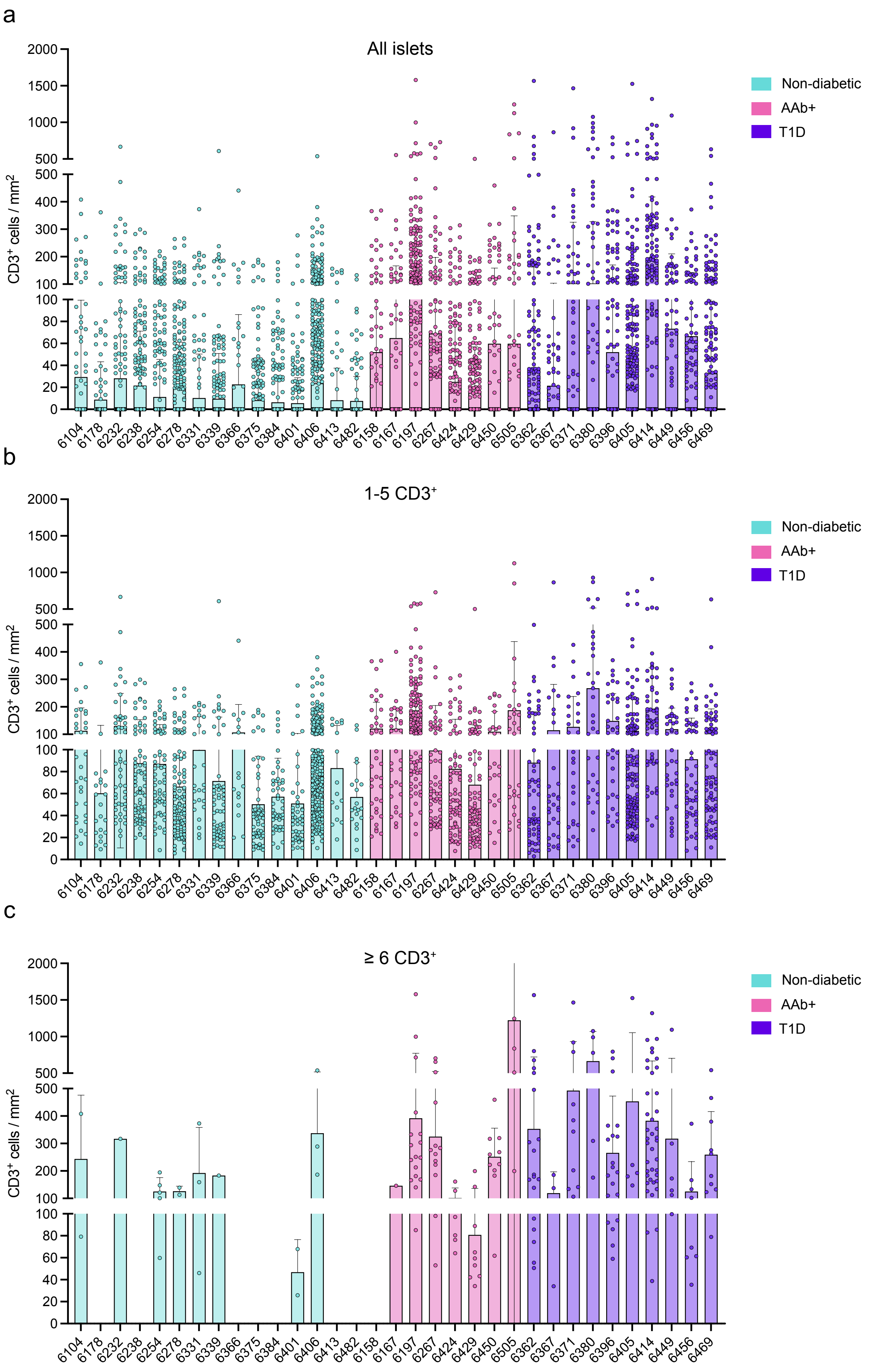

ESM fig. 2: Individual islet T cell density data for each donor. Bar graphs showing the mean + standard deviation of cell densities for all non-diabetic (blue), AAb+ (pink), and T1D (purple) donors. Each dot represents an islet. Data are summarized for (a) all islets, (b) islets infiltrated by 1-5 $CD3^+$ cells, and (c) islets infiltrated by ≥6 $CD3^+$ cells.

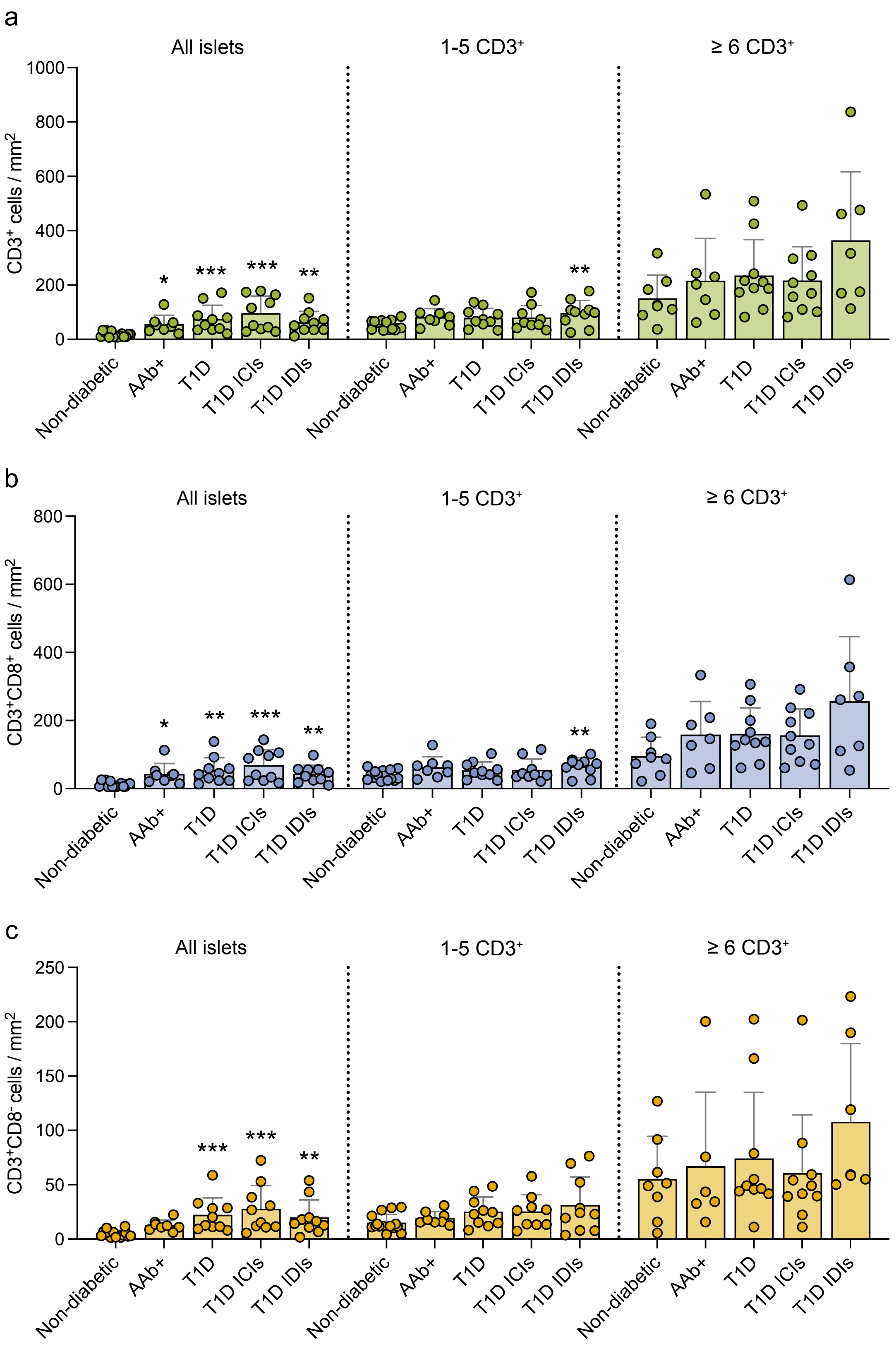

ESM fig. 3: Summarized data showing T cell density for each group. Bar graphs showing the mean + standard deviation of cell densities for the non-diabetic, AAb+, and T1D groups, and for ICIs, and IDIs from T1D donors for all islets, islets infiltrated by 1-5 cells, and islets infiltrated by ≥6 cells, for (a) $CD3^+$ cells (green), (b) $CD3^+CD8^+$ cells (blue), and (c) $CD3^+CD8^-$ cells (orange). Kruskal–Wallis test in a-c was performed. *p*-values corrected for multiple comparisons using Dunn's procedure. $^*p < 0.05$, $^{**}p < 0.01$ and $^{***}p < 0.001$.
